# Preoperative neutrophil-to-lymphocyte ratio and systemic immune-inflammation index as prognostic biomarkers for postoperative pneumonia and pulmonary complications after thoracic surgery: a systematic review and meta-analysis

**DOI:** 10.3389/fmed.2026.1867440

**Published:** 2026-06-05

**Authors:** Xinrui Yin, Shijia Du

**Affiliations:** 1 Department of Anesthesiology, Aerospace Center Hospital, Beijing, China; 2 Department of VIP Dental Service, Peking University Stomatological Hospital, Beijing, China

**Keywords:** neutrophil-to-lymphocyte ratio, postoperative pneumonia, postoperative pulmonary complications, prognostic biomarker, systemic immune-inflammation index, thoracic surgery

## Abstract

**Background:**

Postoperative pneumonia and pulmonary complications are major adverse events following thoracic surgery. The neutrophil-to-lymphocyte ratio (NLR) and systemic immune-inflammation index (SII), two circulating immune-inflammatory biomarkers derived from routine complete blood counts, have been individually associated with postoperative outcomes, but no quantitative synthesis has specifically evaluated their prognostic value for pulmonary outcomes after lung resection or esophagectomy.

**Methods:**

A systematic search of PubMed, Embase, Web of Science, Scopus, CENTRAL, CNKI, and Wanfang Data was conducted from inception to 11 April 2026. Observational studies reporting associations between preoperative NLR and/or SII and postoperative pneumonia or pulmonary complications in adults undergoing lung resection or esophagectomy were eligible. A hierarchical framework prioritized continuous associations as the primary synthesis, with categorical high-versus-low comparisons as secondary. Random-effects models with restricted maximum likelihood estimation and the Hartung–Knapp adjustment were used, and certainty of evidence was assessed using GRADE.

**Results:**

Eight retrospective cohort studies comprising 3,936 patients were included. Higher preoperative NLR and SII showed directionally consistent associations with increased postoperative pulmonary risk across all analyses. For NLR, continuous analyses suggested an approximately twofold increase in the odds of postoperative pneumonia per one-unit increment, and categorical analyses an approximately fourfold increase above study-specific thresholds. For SII, the continuous association was more modest, while categorical analyses indicated more than twice the odds above study-specific cut-offs. However, Hartung–Knapp confidence intervals crossed unity in three of the four pooled analyses, reflecting the very small number of contributing studies (k = 2–3 per analysis), and the overall certainty of evidence was low to very low.

**Conclusion:**

Higher preoperative NLR and SII may be associated with an increased risk of postoperative pneumonia and related pulmonary complications after thoracic surgery, but the evidence base remains small, exclusively retrospective, and geographically concentrated. These low-cost immune-inflammatory indices should be regarded as promising, hypothesis-generating prognostic markers that require confirmation in larger, prospective, and methodologically standardized studies before they can be considered for routine perioperative risk stratification.

**Systematic review registration:**

https://www.crd.york.ac.uk/PROSPERO/view/CRD420261329394, identifier PROSPERO (CRD420261329394).

## Introduction

Postoperative pulmonary complications, particularly postoperative pneumonia, are among the most clinically consequential adverse events following thoracic surgery (1, 2). In patients undergoing lung resection or esophagectomy, these complications substantially increase intensive care unit admission rates, prolong hospitalization, delay subsequent oncologic treatment, and elevate perioperative mortality (1, 3, 4). The heightened vulnerability of thoracic surgical patients to infectious pulmonary outcomes reflects the convergence of multiple predisposing factors, including direct surgical manipulation of the lung parenchyma, the need for one-lung ventilation, impaired postoperative cough and mucociliary clearance, and—in esophagectomy cohorts—additional risks of aspiration and anastomotic-related inflammatory stress (2, 5). Although established risk factors such as age, smoking history, chronic obstructive pulmonary disease, pulmonary function impairment, and surgical extent are incorporated into existing perioperative risk assessment frameworks (3, 6), they do not fully account for interindividual variability in postoperative infectious susceptibility. In particular, the preoperative host immune-inflammatory state—a potentially informative and readily assessable dimension of surgical risk—remains underrepresented in current stratification tools, highlighting the need for simple, low-cost, blood-based biomarkers that may complement conventional clinical risk assessment (7).

The neutrophil-to-lymphocyte ratio (NLR) and the systemic immune-inflammation index (SII) have emerged as candidate circulating immune-inflammatory biomarkers with biological plausibility for predicting infectious pulmonary outcomes (8, 9). NLR reflects the balance between neutrophil-driven innate pro-inflammatory activity and lymphocyte-mediated adaptive immune surveillance (8), while SII incorporates platelet count to additionally capture platelet-driven thromboinflammation—a pathway implicated in endothelial activation, microvascular injury, and amplification of perioperative tissue damage (9, 10). Both indices are derived from routine preoperative complete blood counts, requiring no additional laboratory costs or specialized equipment (8). These indices belong to a broader family of systemic inflammation-based biomarkers—including the C-reactive protein-to-albumin ratio, the systemic inflammation response index, and platelet-based composite indices—that have been increasingly investigated for perioperative risk stratification and dynamic risk assessment in thoracic surgery (11, 12). A growing number of single-center studies, predominantly from Chinese-speaking East Asian thoracic surgical settings, have reported associations between elevated preoperative NLR or SII and postoperative pneumonia or pulmonary complications (13–17). However, the existing evidence remains fragmented by small sample sizes, heterogeneous outcome definitions, variable depth of multivariable adjustment, and inconsistent biomarker parameterization. In particular, studies vary widely in dichotomization thresholds and in the use of continuous versus categorical exposure modeling (13–17). To date, there has been no quantitative synthesis, to our knowledge, specifically focused on the prognostic value of these immune-inflammatory indices for pulmonary outcomes in the thoracic surgical setting.

The present study therefore aims to systematically evaluate and quantitatively synthesize the associations of preoperative NLR and SII with postoperative pneumonia and pulmonary complications after lung resection or esophagectomy. A hierarchical analytical framework was employed, prioritizing continuous biomarker associations as the primary synthesis layer to circumvent the well-recognized problem of cut-off heterogeneity, with categorical high-versus-low comparisons analyzed as a secondary layer. Given that the available evidence was anticipated to be limited in volume and derived predominantly from retrospective studies, this prognostic factor systematic review was conceived as an exploratory, hypothesis-generating synthesis rather than an attempt to establish clinically definitive estimates. It was designed to address three questions: whether preoperative NLR and SII are associated with increased risk of infectious pulmonary outcomes after thoracic surgery, whether such associations are directionally consistent across continuous and categorical analytical frameworks, and whether the current evidence base provides a sufficiently consistent signal to inform future prospective evaluation of their role in perioperative risk stratification.

## Materials and methods

### Protocol, registration, and reporting standards

This prognostic factor systematic review and meta-analysis (PF-SR/MA) evaluates the associations of preoperative neutrophil-to-lymphocyte ratio (NLR) and systemic immune-inflammation index (SII)—two circulating immune-inflammatory biomarkers reflecting the balance between pro-inflammatory neutrophil activation, adaptive lymphocyte-mediated immunity, and platelet-driven thromboinflammation—with infectious pulmonary outcomes after thoracic surgery. The protocol was prospectively registered with PROSPERO (CRD420261329394; registered 1 March 2026). This review is reported in accordance with the Preferred Reporting Items for Systematic Reviews and Meta-Analyses (PRISMA) 2020 statement (18) and follows current best-practice recommendations for prognostic factor evidence synthesis (19). Of note, this study synthesizes prognostic associations rather than diagnostic test accuracy; any predictive performance data (e.g., area under the receiver operating characteristic curve [AUC]) are treated as supplementary and are not conflated with the primary association analyses. Accordingly, the present review estimates the strength and consistency of biomarker–outcome associations and does not evaluate or validate any multivariable prediction model, biomarker-based classification rule, or clinical decision threshold.

### Eligibility criteria

Studies were evaluated using the Population–Exposure–Comparator–Outcome (PECO) framework for prognostic factor research (20).

#### Population

Adults (≥18 years) who underwent elective lung resection (lobectomy, segmentectomy, pneumonectomy, or wedge resection; video-assisted thoracoscopic surgery [VATS] or open thoracotomy) or esophagectomy for malignant or benign indications. Studies enrolling fewer than 30 participants, emergency procedures, and isolated cardiac surgery without a thoracic component were excluded.

#### Exposure (prognostic factors)

Preoperative NLR (neutrophil count ÷ lymphocyte count) and/or SII (platelet count × neutrophil count ÷ lymphocyte count), derived from a routine complete blood count obtained within 30 days before surgery. The exact formula, measurement timing, and units were recorded verbatim for each study.

#### Comparator

For continuous analyses, lower biomarker values served as the implicit reference. For categorical analyses, the low-value group defined by each study’s own cut-off served as the comparator. Because categorical thresholds were derived independently by each study, they were not assumed to be directly comparable or transferable across populations.

#### Outcomes

The primary outcomes were postoperative pneumonia—considered the principal infectious pulmonary outcome—and a composite of postoperative pulmonary complications (PPCs). Postoperative pneumonia was defined as occurring during the index hospitalization (preferred) or within the closest reported time window up to 30 days; the diagnostic criteria and time window were recorded verbatim for each study. The PPCs composite was accepted as defined by the original investigators; the specific components (e.g., pneumonia, atelectasis, acute respiratory distress syndrome, respiratory failure, reintubation, prolonged mechanical ventilation) and time windows were recorded, and heterogeneity by component composition was explored where feasible—specifically by stratifying studies according to whether pneumonia was included as a component and by outcome time window. Studies reporting broader postoperative complication composites that included pulmonary infection or pulmonary complications as a component, but also incorporated non-pulmonary events, were retained as supportive evidence and could contribute to secondary, exploratory quantitative analyses when pulmonary-only data were unavailable or too sparse; such studies were explicitly not included in the primary pulmonary-specific quantitative pooling. Secondary outcomes included 30-day or in-hospital mortality, intensive care unit (ICU) admission and length of stay, total hospital length of stay, reintubation, and duration of mechanical ventilation.

#### Study designs

Prospective and retrospective observational cohort studies and nested case–control studies were eligible. Case reports, case series with fewer than 30 participants, narrative reviews, editorials, and randomized controlled trials were excluded.

### Information sources, search strategy, and study selection

A systematic search was conducted from database inception to 11 April 2026 across PubMed/MEDLINE, Embase (Embase.com), Web of Science Core Collection, Scopus, and the Cochrane Central Register of Controlled Trials (CENTRAL; searched for completeness because it may index conference records or reports relevant to thoracic perioperative outcomes). To capture potentially relevant Chinese-language evidence, the China National Knowledge Infrastructure (CNKI) and Wanfang Data were searched using equivalent Chinese-language terms. Reference lists of all included studies and relevant systematic reviews were hand-searched. No restrictions on publication date, language, or country of origin were applied. The complete electronic search strategies for all databases, including the Chinese-language terms used for CNKI and Wanfang Data, are provided in Supplementary material S1.

The CNKI and Wanfang searches retrieved no records using the predefined Chinese-language search strategy. This finding likely reflects the specific eligibility requirements of the present review, which required preoperative NLR or SII to be evaluated as a prognostic factor for postoperative pneumonia or pulmonary complications after lung resection or esophagectomy, rather than the absence of Chinese clinical interest in inflammation-based biomarkers more broadly. All eligible studies were ultimately identified through non-Chinese bibliographic databases.

Titles and abstracts were screened independently by two reviewers; potentially eligible records underwent full-text assessment against the predefined PECO criteria. Disagreements at either stage were resolved through discussion and consensus. Reasons for full-text exclusion were documented and presented in the PRISMA flow diagram.

### Data extraction and quality control

Two reviewers independently extracted data using a standardized, piloted form; discrepancies were resolved through discussion and consensus. For any Chinese-language records requiring full-text assessment or extraction, data were checked by a bilingual reviewer to ensure translation accuracy and extraction consistency.

The following data were extracted: (i) study characteristics—first author, publication year, country, study design, recruitment period, sample size, follow-up window, funding source, and conflict-of-interest declarations; (ii) patient and surgical characteristics—age, sex, body mass index, smoking history, comorbidities (chronic obstructive pulmonary disease [COPD], diabetes mellitus, cardiovascular disease), pulmonary function (forced expiratory volume in one second [FEV₁] percent predicted, diffusing capacity of the lung for carbon monoxide [DLCO], where reported), surgical approach (VATS versus open), type and extent of resection, proportion with malignant disease, proportion who received neoadjuvant therapy, and American Society of Anesthesiologists physical status distribution; (iii) biomarker measurement details—timing of sampling (days preoperatively), formula, source (routine clinical complete blood count versus research-specific sampling), cut-off value and derivation method (receiver operating characteristic [ROC]/Youden index, median split, or fixed literature-based threshold), and descriptive statistics of the biomarker distribution; (iv) effect estimates—type (odds ratio [OR], hazard ratio [HR], or risk ratio [RR]), point estimate with 95% confidence interval, univariable versus multivariable analysis, and the complete covariate list for each multivariable model (recorded verbatim).

When a study reported multiple adjustment models for the same outcome, the most fully adjusted estimate was preferentially extracted. When a study reported the same outcome across multiple time windows, the in-hospital estimate was preferred, followed by the closest window not exceeding 30 days; alternative time-window estimates were reserved for sensitivity analysis. When a study reported subgroup-specific but not overall estimates for a given outcome, mutually exclusive and statistically independent subgroups were combined within the study using a fixed-effect model before entering the meta-analysis; where subgroups could not be validly combined, the most clinically representative subgroup was selected and the rationale documented. Both continuous and categorical effect estimates were extracted from the same study when available, but were used exclusively in their respective analytical layers to avoid duplicate weighting.

Because multiple publications from the same institution with overlapping recruitment periods may draw on the same patient cohort, prespecified deduplication rules were applied. Studies from the same institution with overlapping data-collection periods were flagged as potentially overlapping; the study with the largest sample size was retained; when sample sizes were equal, the study with the most complete multivariable adjustment was preferred; when both criteria were equal, the most recently published study was retained. All potential overlapping-cohort assessments and related decisions were recorded in the study database and summarized in the Supplementary material when applicable.

### Risk of bias assessment

The risk of bias in each included study was assessed using the Quality In Prognosis Studies (QUIPS) tool (21), which evaluates six domains: study participation, study attrition, prognostic factor measurement, outcome measurement, study confounding, and statistical analysis and reporting. Two reviewers independently applied QUIPS; disagreements were resolved through discussion and consensus.

For the study confounding domain, adequate adjustment was defined *a priori* as a multivariable model including at least three core clinical covariates: age, smoking status or COPD, and surgical approach or extent of resection. The inclusion of additional clinically important covariates—such as tumor stage, pulmonary function parameters (FEV₁ percent predicted, DLCO), nutritional status, diabetes, and cardiovascular comorbidity—was recorded for each study and used to stratify the level of adjustment in sensitivity analyses.

### Effect measures, unit harmonization, and hierarchy of analyses

The analytical framework was organized into three hierarchical layers to address the well-recognized heterogeneity in cut-off values across studies of circulating immune-inflammation biomarkers. All effect estimates were oriented so that values greater than 1 indicated higher risk of the outcome per higher biomarker level (or for the high versus low category), ensuring directional consistency across studies.

#### Primary synthesis: continuous associations

The primary analysis pooled effect estimates expressed per one-unit increment in NLR or per a standardized increment in SII. Because SII values span a wide numerical range and different studies may report effects per 1, per 100, or per 1,000, all SII-related continuous estimates were rescaled to a common increment (per 10^2^ units) for comparability across studies where the original data permitted; this choice was methodological, intended to yield interpretable effect sizes without excessively small or large coefficients, and does not imply a clinically optimal or recommended threshold. When rescaling was not possible, the original increment was retained and the estimate was analyzed in a separate stratum or described narratively, with all assumptions documented. For NLR, a per one-unit increment was the default; estimates reported using a different increment (e.g., per 0.1) were rescaled accordingly. Estimates expressed per one standard deviation were analyzed in a separate stratum and were not combined with per-unit estimates.

#### Secondary synthesis: categorical associations

Studies reporting high-versus-low categories were pooled in a secondary analysis, stratified where feasible by cut-off derivation method (ROC/Youden index, median split, or fixed literature-based threshold) to account for expected heterogeneity introduced by different dichotomization approaches. Because each categorical cut-off was derived independently within its source study and was not externally validated, the resulting pooled estimates were interpreted as exploratory and were not used to endorse any specific threshold for clinical application.

#### No cross-type pooling of effect measures

ORs, HRs, and RRs were pooled within their respective metric only; no conversion across effect-measure types was performed. When a given outcome was reported using more than one metric across studies, parallel meta-analyses were conducted and results compared narratively. If an original publication labeled an effect estimate inconsistently with the statistical model actually used (e.g., an estimate reported as a hazard ratio despite being derived from logistic regression), the estimate was reclassified according to the underlying model type; such decisions were documented explicitly in the data extraction table.

### Data synthesis and statistical analysis

Effect estimates were analyzed on the natural logarithm scale; standard errors were derived from 95% confidence intervals. When published confidence intervals were internally inconsistent with the reported point estimate on the log scale (suggesting probable typographical errors), or when the reported interval lacked sufficient decimal precision for reliable standard error derivation, standard errors were reconstructed from the statistically coherent confidence bound or the reported *p*-value, with all corrections documented in the Supplementary material. The primary pooling model was a random-effects model estimated by restricted maximum likelihood (REML) with the Hartung–Knapp adjustment for the confidence interval of the summary effect (22). As robustness checks, the DerSimonian–Laird random-effects estimator and a fixed-effect (inverse-variance) model were also fitted (23).

Between-study heterogeneity was quantified using the I^2^ statistic (24), the between-study variance τ^2^, and Cochran’s Q test; I^2^ exceeding 50% was interpreted as indicating substantial heterogeneity. Because the number of studies available for each analysis was small, heterogeneity statistics—particularly I^2^ and τ^2^—were themselves estimated with considerable uncertainty and were interpreted descriptively rather than as precise quantities. Random-effects meta-regression was planned for analyses including at least 10 studies, to reduce the risk of spurious findings from limited data; prespecified moderator variables included country (mainland China versus other), surgery type (lung resection versus esophagectomy), surgical approach (VATS versus open), cut-off derivation method, level of multivariable adjustment, and neoadjuvant therapy exposure (>50% versus ≤50%).

*Subgroup analyses.* The following subgroups were prespecified: (i) lung resection versus esophagectomy, (ii) VATS versus open surgery, (iii) mainland China versus other settings, (iv) cut-off derivation method, (v) level of adjustment (multivariable-adjusted versus univariable/unadjusted), and (vi) neoadjuvant therapy exposure. Because the number of contributing studies was insufficient, prespecified subgroup comparisons and meta-regression were not formally performed; subgroup-related findings were therefore summarized descriptively where relevant.

#### Sensitivity analyses

Prespecified sensitivity analyses included: (a) excluding studies rated as high risk of bias on QUIPS, (b) restricting to multivariable-adjusted estimates only, (c) restricting to prospective cohort designs, (d) excluding studies with neoadjuvant therapy exposure exceeding 50%, (e) restricting to lung resection studies only, and (f) excluding studies flagged during overlapping-cohort adjudication. Sensitivity analyses were performed only when at least two studies remained available for comparison; otherwise, the intended analysis was noted as not feasible.

#### Supplementary analysis of predictive performance

When at least three studies reported AUC values for the same biomarker–outcome pair—a minimum chosen to avoid unstable summary estimates from sparse data—summary AUC estimates were derived using a random-effects model on the logit-transformed AUC scale, stratified by validation type (apparent performance, internal validation, or external validation) to account for the upward bias inherent in non-validated estimates. When fewer than three studies reported AUC, results were summarized narratively with explicit acknowledgment of overfitting risk. These AUC summaries, if available, were treated strictly as supplementary descriptions of discrimination and were not used to validate any prediction model, biomarker-based classification rule, or clinical decision threshold.

#### Publication bias

For each meta-analysis including 10 or more studies, funnel plot asymmetry was visually inspected and formally tested using Egger’s regression test (25); trim-and-fill analysis was conducted as an exploratory analysis and interpreted cautiously (26). When fewer than 10 studies were available, formal assessment of publication bias was not performed, and its limited feasibility was explicitly acknowledged.

All statistical analyses were performed in R (version 4.4.2) using the meta (version 7.0–0) and metafor (version 4.6–0) packages; Stata was available as a secondary analysis platform.

### Certainty of evidence

The certainty of the body of evidence for each main biomarker–outcome analysis was evaluated using the Grading of Recommendations, Assessment, Development and Evaluations (GRADE) framework adapted for prognostic factor research (27), considering risk of bias (informed by QUIPS ratings), inconsistency, indirectness, imprecision, and publication bias. Consistent with GRADE guidance for prognostic factor evidence, the body of evidence from observational prognostic studies was not automatically rated down for observational design alone but was assessed across the five domains above, with particular attention to imprecision given the small number of studies per analysis. Evidence certainty was classified as high, moderate, low, or very low. The certainty ratings for each main analysis, together with the corresponding pooled estimates and the main reasons for downgrading, are presented in a Summary of Findings table and summarized in the Results.

## Results

### Study selection

**Figure 1:**
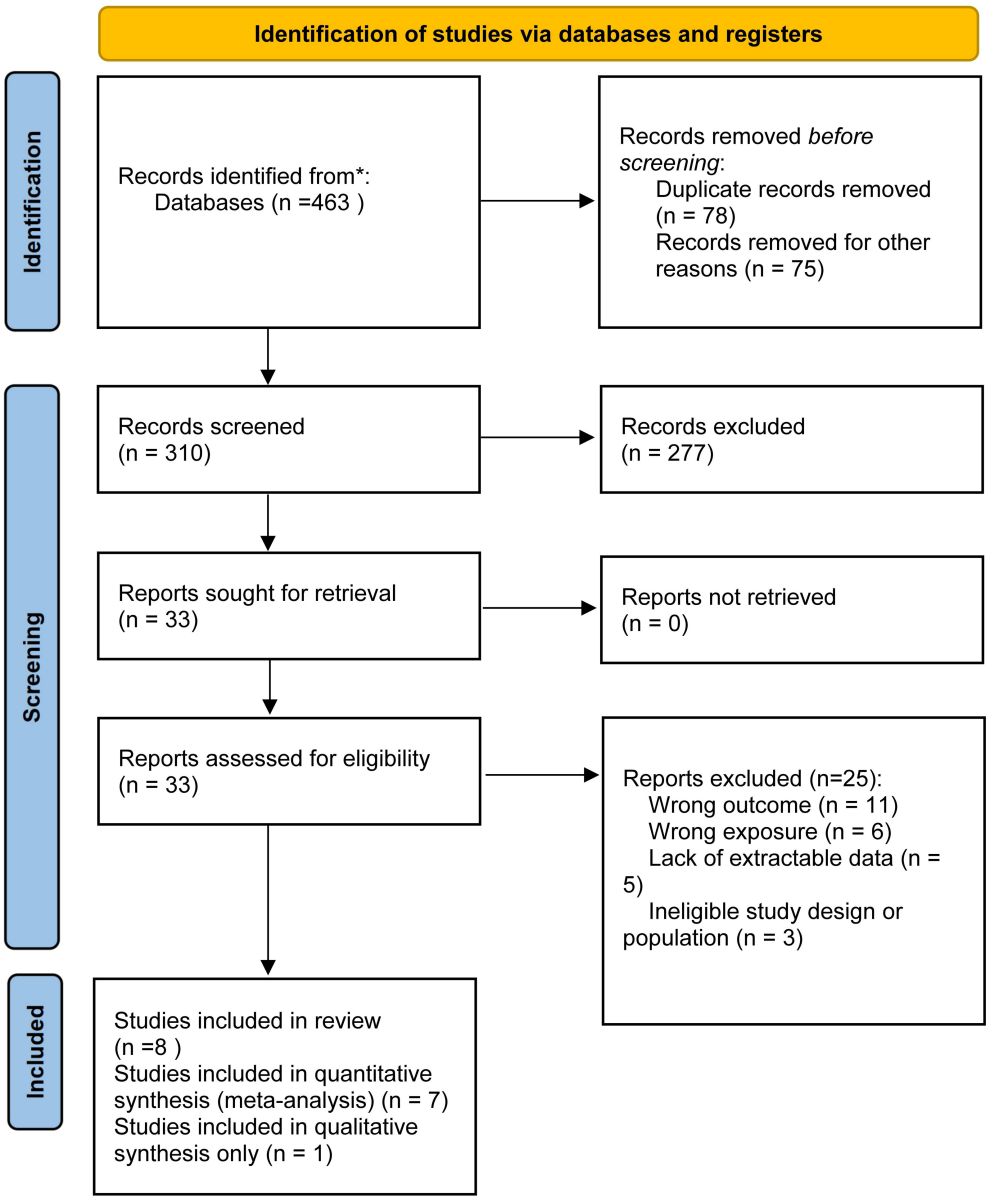
PRISMA 2020 flow diagram of study identification and selection. Seven databases were searched from inception to 11 April 2026: PubMed (*n* = 110), Embase (*n* = 115), Scopus (*n* = 199), Web of Science (*n* = 17), CENTRAL (*n* = 22), CNKI (*n* = 0), and Wanfang Data (*n* = 0). Eight studies met the eligibility criteria; seven contributed to quantitative synthesis and one to qualitative synthesis only.

The database search identified 463 records: 110 from PubMed, 115 from Embase, 199 from Scopus, 17 from Web of Science Core Collection, and 22 from CENTRAL. No records were retrieved from CNKI or Wanfang Data. Hand-searching of reference lists yielded no additional eligible studies. After removal of 78 duplicate records and 75 records excluded before screening for other reasons, 310 records underwent title and abstract screening. Of these, 33 reports were sought for retrieval and assessed in full text. Twenty-five reports were excluded, primarily because of wrong outcome (*n* = 11), wrong exposure (*n* = 6), lack of extractable data (*n* = 5), or ineligible study design or population (*n* = 3). Ultimately, eight studies were included in the review (13–17, 28–30). Seven contributed to the quantitative synthesis (meta-analysis) and one was included in the qualitative synthesis only. The study selection process is presented in Figure 1.

### Characteristics of included studies

The characteristics of the eight included studies are summarized in Table 1. All were retrospective cohort studies. Six were conducted in mainland China, one in Taiwan, and one in France. Sample sizes ranged from 111 to 1,486 participants, with a total of 3,936 patients across all studies.

**Table 1:** Characteristics of included studies evaluating preoperative NLR and/or SII as prognostic factors for postoperative pneumonia or pulmonary complications after lung resection or esophagectomy.

Study	Country	Design	Population/surgery	*N*	Preoperative treatment	Biomarker	Exposure format	Cut-off	Outcome	Outcome window	Effect estimate used	Adjustment status
Miao (13)	China	Retrospective cohort	Lung cancer undergoing VATS segmentectomy/lobectomy	386	None =/NR	SII	Continuous + categorical	261	Postoperative pneumonia	Within 7 days	Adjusted OR	Adjusted
Lo (14)	Taiwan	Retrospective cohort	Stage III ESCC undergoing esophagectomy after chemoradiotherapy	111	CRT	NLR	Categorical	3	PPCs	Within 30 days	Adjusted OR	Adjusted
Shi (45)	China	Retrospective cohort	Esophageal cancer undergoing esophagectomy	171	None / NR	NLR	Categorical	2.30	Study-defined postoperative complications (pulmonary infection included; mixed pulmonary/non-pulmonary composite)	In-hospital	Adjusted OR	Adjusted
Shen (15)	China	Retrospective cohort	NSCLC undergoing lobectomy	1,058	None / NR	NLR	Continuous	N/A	Postoperative pneumonia	Within 30 days	Adjusted OR	Adjusted
Ding (16)	China	Retrospective cohort	ESCC undergoing McKeown esophagectomy after neoadjuvant chemo-immunotherapy	312	Chemo-immunotherapy	NLR + SII	Continuous	N/A	Postoperative pneumonia	Within 30 days	Adjusted OR	Adjusted
Xiaowei (46)	China	Retrospective cohort	Lung cancer undergoing resection	204	None / NR	SII	Categorical	320.22	PPCs	Within 1 month	Adjusted OR	Adjusted
de Fréminville (30)	France	Retrospective cohort	NSCLC undergoing lobectomy	208	None / NR	NLR + SII	Continuous	N/A	Study-defined major complications	In-hospital	Unadjusted OR	Unadjusted
Jiang (17)	China	Retrospective cohort	NSCLC undergoing pulmonary resection	1,486	None / NR	SII	Continuous	N/A	Postoperative pneumonia	Within 30 days	Adjusted OR	Adjusted

VATS, video-assisted thoracoscopic surgery; ESCC, esophageal squamous cell carcinoma; CRT, chemoradiotherapy; NSCLC, non-small cell lung cancer; NLR, neutrophil-to-lymphocyte ratio; SII, systemic immune-inflammation index; PPCs, postoperative pulmonary complications; OR, odds ratio; NR, not reported; N/A, not applicable. Cut-off values are shown for studies using categorical exposure modeling; “N/A” indicates continuous-only analyses. For Miao (13), which reported both continuous and categorical analyses, the adjusted OR from the continuous model was prioritized for the primary quantitative synthesis. Lo et al. (14) reported the effect estimate as a hazard ratio in the original publication; however, the underlying analysis was logistic regression, and the estimate was treated as an odds ratio. Effect estimates from de Fréminville (30) were excluded from the primary meta-analysis due to a lack of multivariable adjustment.

In terms of surgical population, five studies evaluated patients undergoing lung resection for non-small cell lung cancer or mixed lung cancer surgical populations, whereas three focused on esophagectomy cohorts with esophageal squamous cell carcinoma. Among the esophageal cancer studies, Lo et al. (14) included patients undergoing surgery after chemoradiotherapy and Ding et al. (16) specifically examined patients treated with neoadjuvant chemo-immunotherapy before McKeown esophagectomy, indicating clinical heterogeneity in preoperative oncologic treatment exposure. The remaining six studies either explicitly reported no neoadjuvant treatment or did not provide sufficient detail on preoperative oncologic therapy.

Regarding prognostic biomarkers, three studies evaluated preoperative SII only (13, 17, 46), three evaluated preoperative NLR only (14, 15, 45), and two assessed both NLR and SII (16, 30). Continuous biomarker analyses were reported in five studies and categorical high-versus-low comparisons in four studies, with one study (13) providing both; not all reported estimates were eligible for the primary quantitative synthesis. Among the four studies using categorical exposure modeling, all cut-off values were derived from ROC/Youden index analysis: SII cut-offs were 261 (13) and 320.22 (46), and NLR cut-offs were 2.30 (45) and 3.0 (14). Because all four cut-offs were derived through study-specific ROC/Youden optimization, no variation in derivation method was available across studies. All studies measured biomarkers from routine preoperative complete blood counts obtained within 30 days before surgery.

Outcome definitions varied across studies. Four studies used postoperative pneumonia as the primary outcome (13, 15–17), two assessed postoperative pulmonary complications using pulmonary-specific composite definitions (14; 46), and two reported broader study-defined postoperative complications that included pulmonary infection but also incorporated non-pulmonary events (45; 30). Outcome time windows ranged from within 7 days to 30 days (or 1 month) after surgery; two studies used in-hospital events as the assessment window. This variability in outcome definition—spanning pneumonia-specific endpoints, pulmonary-specific composites, and broader mixed complication composites—was a key source of clinical heterogeneity and directly informed the prespecified hierarchical synthesis, in which only pulmonary-specific outcomes contributed to the primary pooling and broader composites were restricted to secondary, exploratory or supportive analyses.

Seven studies reported multivariable-adjusted odds ratios derived from logistic regression models. Lo et al. (14) reported the effect estimate as a hazard ratio in the original publication; however, the underlying analysis was logistic regression, and the estimate was therefore reclassified as an odds ratio in the present synthesis in accordance with the prespecified rule for effect-measure reclassification. One study (30) contributed unadjusted estimates only, as the inflammatory biomarkers did not reach statistical significance in the final multivariable model and were therefore not retained. Overall, the included evidence base was characterized by heterogeneity in surgical setting, immune-inflammatory biomarker parameterization, preoperative oncologic treatment exposure, and outcome definition, which informed the prespecified stratified synthesis strategy.

### Risk of bias assessment

**Figure 2:**
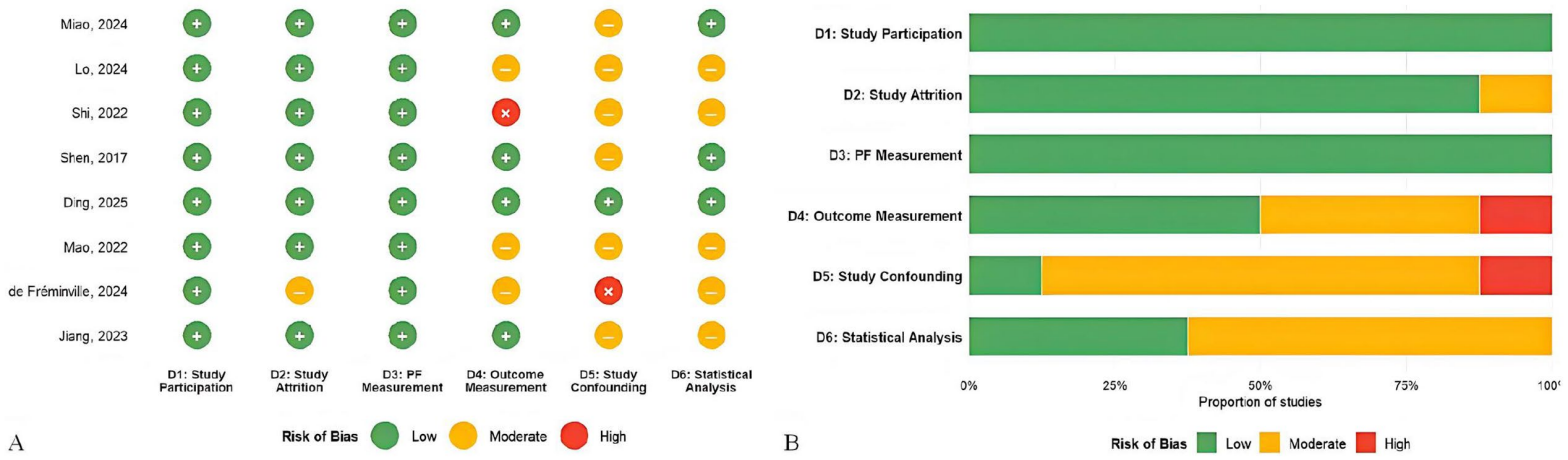
Risk of bias assessment using the Quality In Prognosis Studies (QUIPS) tool. **(A)** Traffic-light plot showing domain-level judgments for each included study. **(B)** Summary plot showing the proportion of studies rated as low, moderate, or high risk of bias within each domain. Green indicates low risk, amber moderate risk, and red high risk of bias.

Risk of bias was assessed using the Quality In Prognosis Studies (QUIPS) tool across six domains. The domain-level judgments for each study are presented in Figure 2 (Panel A, traffic-light plot; Panel B, summary plot), with detailed rationales provided in Supplementary Table S1. Overall, one study (16) had the most favorable QUIPS profile, six studies were rated as moderate risk in at least one domain, and one study (30) was rated as high risk in the confounding domain and moderate risk in the attrition domain.

Study participation and prognostic factor measurement were rated as low risk of bias in all eight studies. All studies enrolled clearly defined surgical populations with reasonable eligibility criteria and measured NLR and/or SII from routine preoperative complete blood counts using standard formulas with documented sampling timing. Study attrition was rated as low risk in seven studies that assessed short-term postoperative outcomes (within 7 to 30 days) with minimal loss to follow-up; de Fréminville (30) was rated as moderate risk because a substantial proportion of otherwise eligible patients were excluded from the final analysis owing to missing perioperative hematologic data.

The main methodological concerns arose in the remaining three domains. For outcome measurement, four studies were rated as low risk based on explicit pneumonia diagnostic criteria with defined time windows, two as moderate risk because they used composite pulmonary complication endpoints rather than isolated postoperative pneumonia (14, 46), one as moderate risk because it used a broader major-complication composite including both pulmonary and non-pulmonary events (30), and one as high risk (45) because the outcome combined pulmonary infection with anastomotic leakage, venous thromboembolism, and other unrelated complications. Study confounding was the most common source of concern: six studies were rated as moderate risk because multivariable models omitted one or more prespecified core covariate domains—age, smoking status/COPD, and surgical approach/extent of resection; one study (16) was judged as comparatively lower risk in the confounding domain because it adjusted for the ARISCAT score, which captures several clinically relevant perioperative pulmonary risk dimensions including age, preoperative SpO₂, and respiratory infection history, although direct one-to-one correspondence with all prespecified core covariates was not always explicit; and one was rated as high risk (30) because inflammatory biomarkers were not retained in the final multivariable model. For statistical analysis and reporting, five studies were rated as moderate risk owing to issues such as mislabeling of effect measures (14), limited justification for cut-off selection, or use of raw SII units yielding effect estimates of limited clinical interpretability (17); the remaining three studies were rated as low risk.

### Primary synthesis: continuous associations

**Figure 3:**
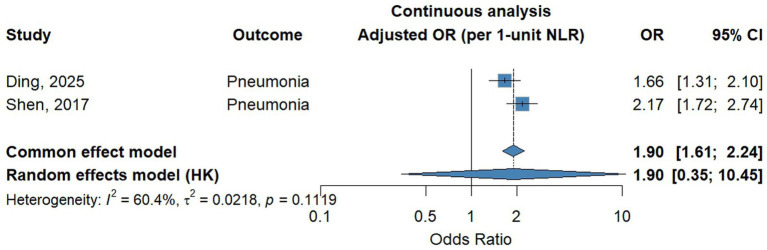
Forest plot of the continuous association between preoperative NLR (per 1-unit increment) and postoperative pneumonia after thoracic surgery. Effect estimates are multivariable-adjusted odds ratios. The pooled estimate was calculated using a random-effects model (REML) with the Hartung–Knapp adjustment.

**Figure 4:**
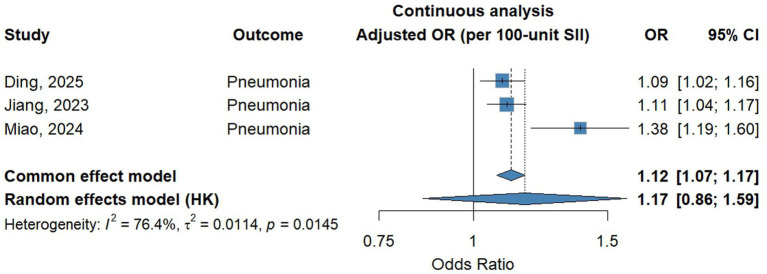
Forest plot of the continuous association between preoperative SII (per 10^2^ units) and postoperative pneumonia after thoracic surgery. Effect estimates are multivariable-adjusted odds ratios. Standard errors for Miao (13) and Ding (16) were derived from the lower confidence limit due to probable typographical errors in the published upper bounds (see text). The effect estimate for Jiang (17) was rescaled from per-unit to per 100-unit increment. The pooled estimate was calculated using a random-effects model (REML) with the Hartung–Knapp adjustment.

The primary quantitative synthesis focused on continuous biomarker associations with postoperative pneumonia, because continuous-effect estimates for pulmonary-specific PPC composites were too sparse for comparable primary pooling. Multivariable-adjusted odds ratios were pooled using random-effects models with REML estimation and the Hartung–Knapp adjustment. Results are summarized in Table 2 and presented as forest plots in Figures 3, 4.

**Table 2:** Summary of primary and secondary meta-analytic results for associations between preoperative immune-inflammatory biomarkers and postoperative pneumonia or pulmonary complications after thoracic surgery.

Biomarker	Unit/Category	Outcome	k	*N*	Pooled OR (common effect) [95% CI]	Pooled OR (random effects, HK) [95% CI]	I^2^ (%)	τ^2^	Q test *p*-value
Continuous associations (primary layer)									
NLR	per 1-unit	Postoperative pneumonia	2	1,370	1.90 [1.61–2.24]	1.90 [0.35–10.45]	60.4%	0.022	0.11
SII	per 10^2^ units	Postoperative pneumonia	3	2,184	1.12 [1.07–1.17]	1.17 [0.86–1.59]	76.4%	0.011	0.01
Categorical associations (secondary layer)									
NLR	high vs. low	PPCs/complications	2	282	3.98 [1.91–8.31]	3.98 [0.79–19.96]	0.0%	0	0.74
SII	high vs. low	Pneumonia/PPCs	2	590	2.53 [1.42–4.53]	2.53 [1.04–6.17]	0.0%	0	0.81

Effect estimates are multivariable-adjusted odds ratios. The common-effect model uses the inverse-variance method; the random-effects model uses restricted maximum likelihood (REML) estimation with the Hartung–Knapp (HK) adjustment, which produces wider and more conservative confidence intervals, particularly when the number of contributing studies (k) is small. For continuous SII analyses, all estimates were standardized to a per 10^2^ unit increment; standard errors for Miao (13) and Ding (16) were derived from the lower confidence limit due to probable typographical errors in the published upper bounds; the estimate for Jiang (17) was rescaled from per-unit and the standard error derived from the reported *p*-value (see Supplementary Table S2 for details). For categorical analyses, study-specific cut-off values and derivation methods are reported in Table 1. NLR, neutrophil-to-lymphocyte ratio; SII, systemic immune-inflammation index; PPCs, postoperative pulmonary complications; OR, odds ratio; CI, confidence interval; k, number of included studies; N, total sample size.

#### NLR (per 1-unit increment)

Two studies (15, 16) contributed adjusted ORs for the continuous association between preoperative NLR and postoperative pneumonia after thoracic surgery. Both studies reported effect estimates in the same direction, with ORs of 2.17 (95% CI 1.72–2.74) and 1.66 (95% CI 1.31–2.10), respectively. Under the prespecified random-effects model with Hartung–Knapp adjustment, the pooled OR was 1.90 (95% CI 0.35–10.45), with the confidence interval crossing unity. This very wide interval reflects the substantial uncertainty introduced by applying this conservative correction when only two studies are available, despite the directionally consistent estimates observed in the individual studies. The corresponding common-effect pooled OR was 1.90 (95% CI 1.61–2.24). Between-study heterogeneity was moderate (I^2^ = 60.4%, τ^2^ = 0.022, Q test *p* = 0.11), although this estimate is itself imprecise given that only two studies were available. Despite the wide Hartung–Knapp interval, the consistent direction of effect across both studies—spanning lung resection (15) and esophagectomy after neoadjuvant chemo-immunotherapy (16)—suggests a directionally consistent association, with point estimates approximating a twofold increase in the odds of postoperative pneumonia per one-unit increase in preoperative NLR.

#### SII (per 10^2^ units)

Three studies (13, 16, 17) contributed adjusted ORs for the continuous association between preoperative SII and postoperative pneumonia. To enable pooling on a common scale, all effect estimates were standardized to a per 100-unit increment in SII. For Jiang et al. (17), the original per-unit OR (1.001) was rescaled by exponentiating the log-transformed estimate to the power of 100; the standard error was derived from the reported *p*-value (*p* < 0.001) because the published confidence interval lacked sufficient decimal precision for reliable computation. For Miao et al. (13) and Ding et al. (16), the published upper confidence limits were statistically inconsistent with the point estimates and lower limits on the log scale, suggesting probable typographical errors in the original publications; standard errors were therefore derived from the lower confidence limits, yielding log-symmetric intervals (corrected ORs: Miao, 1.38 [1.19–1.60]; Ding, 1.09 [1.02–1.16]). The original and corrected values are reported in Supplementary Table S2.

After rescaling and correction, all three studies demonstrated a consistent positive association between higher preoperative SII and postoperative pneumonia, with per 100-unit ORs of 1.38 (13), 1.09 (16), and 1.11 (17). Under the prespecified random-effects model with Hartung–Knapp adjustment, the pooled OR was 1.17 (95% CI 0.86–1.59), with the confidence interval crossing unity. The corresponding common-effect pooled OR was 1.12 (95% CI 1.07–1.17). Between-study heterogeneity was substantial (I^2^ = 76.4%, τ^2^ = 0.011, Q test *p* = 0.01), driven primarily by the larger effect estimate in Miao et al. (13); this heterogeneity, together with the standard-error reconstruction required for two of the three studies, contributes additional uncertainty to the pooled estimate.

#### Supplementary continuous evidence (unadjusted)

One additional study (30) reported unadjusted continuous associations for both NLR (OR 1.25, 95% CI 0.98–1.59, per SD) and SII (OR 1.21, 95% CI 0.96–1.54, per SD). Because these estimates lacked multivariable adjustment and were expressed per standard deviation rather than per unit increment, they were not included in the primary pooled analyses. Both point estimates were directionally consistent with the adjusted findings, although neither reached statistical significance.

#### Summary of primary continuous findings

Across both biomarkers, all individual study estimates pointed in the same direction, suggesting that higher preoperative NLR and SII may be associated with increased risk of postoperative pneumonia following thoracic surgery. The magnitude of association was notably larger for NLR (pooled OR approximately 1.9 per unit) than for SII (pooled OR approximately 1.1–1.2 per 100 units), which is expected given the different numerical scales and biological composition of these indices. Although the Hartung–Knapp confidence intervals crossed unity in both analyses owing to the limited number of contributing studies (k = 2–3), the consistent direction of effect across studies and the concordant common-effect estimates support a directionally consistent prognostic association. Given the very small number of studies, wide Hartung–Knapp intervals, and low certainty of evidence, these findings should be regarded as exploratory and hypothesis-generating and require confirmation in larger prospective datasets.

### Secondary synthesis: categorical associations (high vs. low)

**Figure 5:**
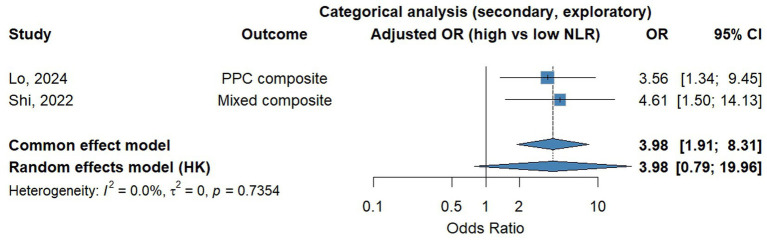
Forest plot of the categorical association between preoperative NLR (high vs. low) and postoperative complications after esophageal surgery. Cut-off values were 3.0 (14) and 2.30 (45), both derived from ROC/Youden index analysis. Effect estimates are multivariable-adjusted odds ratios pooled using a random-effects model (REML) with the Hartung–Knapp adjustment.

**Figure 6:**
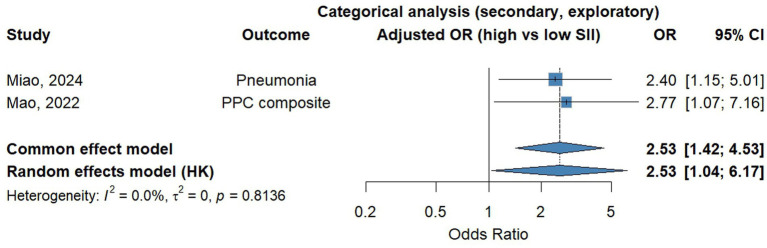
Forest plot of the categorical association between preoperative SII (high vs. low) and postoperative pneumonia or pulmonary complications after lung resection. Cut-off values were 261 (13) and 320.22 (46), both derived from ROC/Youden index analysis. Effect estimates are multivariable-adjusted odds ratios pooled using a random-effects model (REML) with the Hartung–Knapp adjustment.

Categorical analyses were presented for biomarker–outcome pairs with the largest number of contributing studies; remaining categorical results are summarized in Supplementary Table S3. As specified *a priori*, these categorical analyses were considered secondary and exploratory, because all cut-offs were study-specific and not externally validated. Results are shown in Figures 5, 6 and the lower panel of Table 2.

#### NLR (high vs low)

Two studies contributed categorical adjusted ORs for preoperative NLR dichotomized at study-specific cut-offs: Lo et al. (14) used a ROC/Youden index-derived cut-off of 3.0 in an esophagectomy cohort with PPCs as the outcome (OR 3.56, 95% CI 1.34–9.45), and Shi et al. (2022) used a ROC/Youden index-derived cut-off of 2.30 in an esophagectomy cohort with broader study-defined postoperative complications—including non-pulmonary events—as the outcome (OR 4.61, 95% CI 1.50–14.13). Neither categorical analysis showed detectable statistical heterogeneity (I^2^ = 0.0%, τ^2^ = 0, Q test *p* = 0.74), although with only two studies this should not be interpreted as evidence of true homogeneity. Under the prespecified random-effects model with Hartung–Knapp adjustment, the pooled OR was 3.98 (95% CI 0.79–19.96), with the confidence interval crossing unity; the widened interval mainly reflects the conservative penalty inherent to this correction with only two studies and should be interpreted with caution. The corresponding common-effect pooled OR was 3.98 (95% CI 1.91–8.31). The consistently large point estimates in both studies suggest that elevated preoperative NLR may be associated with an approximately fourfold increase in the odds of adverse postoperative outcomes after esophageal surgery; however, this estimate should be interpreted with particular caution because one contributing study (45) used a broader mixed complication composite rather than a pulmonary-specific endpoint, introducing outcome indirectness into the pooled result.

#### SII (high vs low)

Two studies contributed categorical adjusted ORs for preoperative SII: Miao et al. (13) used a ROC/Youden index-derived cut-off of 261 in a lung resection cohort with postoperative pneumonia as the outcome (OR 2.40, 95% CI 1.15–5.01), and Mao et al. (2022) used a ROC/Youden index-derived cut-off of 320.22 in a lung resection cohort with PPCs as the outcome (OR 2.77, 95% CI 1.07–7.16). Neither study contributed detectable statistical heterogeneity (I^2^ = 0.0%, τ^2^ = 0, Q test *p* = 0.81), with the same caveat regarding the small number of studies. Under the prespecified random-effects model with Hartung–Knapp adjustment, the pooled OR was 2.53 (95% CI 1.04–6.17), with the confidence interval remaining above unity—the only analysis in the present review in which the conservative Hartung–Knapp interval did not cross unity. The corresponding common-effect pooled OR was 2.53 (95% CI 1.42–4.53). These findings suggest that patients classified as high SII according to study-specific cut-offs may have more than twice the odds of developing postoperative pneumonia or pulmonary complications after lung resection, although this observation is based on only two studies using non-validated thresholds.

#### Comparison of continuous and categorical findings

The categorical results were directionally consistent with the primary continuous analyses for both biomarkers. The categorical effect sizes were larger in magnitude (NLR: OR ~4.0; SII: OR ~2.5) than the continuous per-unit estimates, which is expected because categorical analyses compare extreme groups rather than evaluating incremental risk; this difference is a recognized statistical consequence of dichotomization and does not indicate a stronger underlying association. Notably, neither categorical analysis showed detectable statistical heterogeneity (I^2^ = 0%), in contrast to the moderate-to-substantial heterogeneity observed in the continuous syntheses. This apparent difference likely reflects the very small number of studies and the more homogeneous surgical populations within each categorical analysis (esophagectomy only for NLR; lung resection only for SII), rather than genuinely greater consistency, and the I^2^ values from these two-study analyses carry substantial uncertainty.

### Robustness and small-study effects

#### Subgroup analyses

With only two to three studies contributing to each meta-analysis, formal subgroup comparisons and meta-regression were not feasible, as prespecified in the Methods (minimum k = 10 for meta-regression). Nevertheless, several observations can be made from the pattern of available evidence. The continuous NLR analysis included one lung resection study (15; OR 2.17) and one esophagectomy study (16; OR 1.66); the categorical NLR analysis comprised two esophagectomy studies exclusively. Conversely, the continuous SII analysis included two lung resection studies (13, 17) and one esophagectomy study (16), while the categorical SII analysis comprised two lung resection studies exclusively. This distribution precluded direct within-analysis comparison of lung resection versus esophagectomy for any biomarker. All four categorical studies used ROC/Youden index-derived cut-offs; therefore, no variation in cut-off derivation method was available for subgroup comparison.

Regarding preoperative oncologic treatment, two of the eight included studies enrolled patients who received neoadjuvant therapy: Lo et al. (14) after chemoradiotherapy and Ding et al. (16) after chemo-immunotherapy. In the continuous NLR analysis, excluding Ding (16) would leave only Shen (15), precluding pooling. In the categorical NLR analysis, excluding Lo (14) would similarly leave only Shi (2022). The influence of neoadjuvant therapy on baseline NLR/SII levels and their prognostic value therefore could not be formally evaluated but remains an important source of clinical heterogeneity.

Seven of the eight included studies were conducted in East Asian settings (six in mainland China, one in Taiwan) and one in France. The sole non-Asian study (30) contributed only unadjusted estimates and was already excluded from the primary synthesis, making geographic subgroup comparison not possible.

#### Sensitivity analyses

Among the six prespecified sensitivity analyses, only a limited subset could be meaningfully conducted given the small number of contributing studies. The results of all prespecified robustness checks are summarized in Table 3.

**Table 3:** Summary of prespecified sensitivity and robustness checks.

Analysis	Modification	k	Pooled OR [95% CI]	I^2^ (%)	Conclusion changed?
Continuous NLR (per 1-unit)	Primary analysis	2	1.90 [1.61–2.24]	60.4	—
	Exclude high QUIPS risk	2	1.90 [1.61–2.24]	60.4	No change (high-risk study already excluded)
	Adjusted estimates only	2	1.90 [1.61–2.24]	60.4	No change (identical to primary)
	Lung resection only	1	Not pooled (Shen, 2017 only)	—	Not feasible
	Exclude neoadjuvant >50%	1	Not pooled (Shen, 2017 only)	—	Not feasible
Continuous SII (per 10^2^ units)	Primary analysis	3	1.12 [1.07–1.17]	76.4	—
	Exclude high QUIPS risk	3	1.12 [1.07–1.17]	76.4	No change (high-risk study already excluded)
	Adjusted estimates only	3	1.12 [1.07–1.17]	76.4	No change (identical to primary)
	Lung resection only	2	1.14 [1.08–1.20]	86.6	Consistent
	Exclude neoadjuvant >50%	2	1.14 [1.08–1.20]	86.6	Consistent
Categorical NLR (high vs low)	Primary analysis	2	3.98 [1.91–8.31]	0.0	—
	Exclude neoadjuvant >50%	1	Not pooled (shi (45) only)	—	Not feasible
Categorical SII (high vs low)	Primary analysis	2	2.53 [1.42–4.53]	0.0	—
All analyses	Prospective cohorts only	0	Not estimable	—	Not applicable (all retrospective)
	Exclude overlapping cohorts	—	—	—	Not applicable (none flagged)

Where formal re-pooling was feasible, common-effect pooled odds ratios are reported for comparability with the primary analysis (Table 2). Several prespecified analyses could not be conducted due to the limited number of contributing studies or because all included studies were retrospective in design. A dash (—) indicates that the analysis was not applicable or not feasible. OR, odds ratio; CI, confidence interval; k, number of included studies; QUIPS, Quality In Prognosis Studies; NLR, neutrophil-to-lymphocyte ratio; SII, systemic immune-inflammation index.

Excluding the study rated as high risk of bias on QUIPS (30) was not informative for the primary pooled analyses because this study had already been excluded from quantitative synthesis owing to the lack of usable multivariable-adjusted estimates. Restriction to multivariable-adjusted estimates only was not informative because all studies contributing to the primary pooled analyses were already multivariable-adjusted. The sensitivity analysis restricting to prospective cohort designs could not be conducted because all eight included studies were retrospective.

Restricting to lung resection studies only was explored for the continuous SII analysis: removing Ding (16), the sole esophagectomy study, left Miao (13) and Jiang (17), yielding a common-effect pooled OR of 1.14 (95% CI 1.08–1.20) with increased heterogeneity (I^2^ = 86.6%), consistent in direction and magnitude with the full three-study result (OR 1.12 [1.07–1.17]). The rise in heterogeneity upon removing Ding (16) suggests that the effect estimate from this esophagectomy study was intermediate between the two lung resection studies, and that the primary source of statistical heterogeneity lies in the differing effect magnitudes between Miao (13) and Jiang (17) rather than in surgical type. For continuous NLR, restricting to lung resection would leave only Shen (15), precluding pooling. For the categorical analyses, NLR high-versus-low comprised esophagectomy studies only and SII high-versus-low comprised lung resection studies only, so further restriction by surgery type was not meaningful.

For continuous SII, excluding the sole study with neoadjuvant therapy exposure exceeding 50% (16) yielded results identical to the lung-resection-only sensitivity analysis (common-effect OR 1.14, 95% CI 1.08–1.20; I^2^ = 86.6%), confirming directional consistency. For continuous and categorical NLR, this exclusion left only a single study in each analysis, precluding pooling.

No included studies met the prespecified criteria for probable overlapping cohorts after adjudication based on institution, recruitment period, and sample size; therefore, this sensitivity analysis was not applicable.

Overall, the primary findings remained directionally stable across all sensitivity scenarios that could be conducted, although the very small number of studies per analysis substantially limits the informativeness of these robustness checks.

#### Publication bias and small-study effects

Formal assessment of publication bias using funnel plots and Egger’s regression test requires a minimum of 10 studies per meta-analysis. As none of the four pooled analyses met this threshold (k ranged from 2 to 3), these tests were not performed. The potential for publication bias cannot be excluded, particularly given the predominance of small-to-moderate sample sizes and the overrepresentation of studies from a single geographic region. This limitation is further addressed in the Discussion.

### Certainty of evidence

The certainty of evidence for the four main biomarker–outcome analyses, assessed using the GRADE framework adapted for prognostic factor research, is summarized in Table 4. Overall certainty was rated as very low for the continuous NLR, continuous SII, and categorical NLR analyses, and low for the categorical SII analysis. The principal reasons for downgrading were serious imprecision arising from the very small number of contributing studies (k = 2–3) and the correspondingly wide Hartung–Knapp confidence intervals, which crossed unity in three of the four analyses; risk-of-bias concerns related to the exclusively retrospective design and variable confounding control across studies; inconsistency, reflected in the moderate-to-substantial heterogeneity observed in the continuous analyses; and indirectness, most notably in the categorical NLR analysis, where one contributing study used a broader mixed complication composite rather than a pulmonary-specific endpoint. Additional uncertainty in the continuous SII analysis arose from the scale harmonization and standard-error reconstruction required for several studies. The categorical SII analysis was rated as low rather than very low because both contributing studies originated from anatomically homogeneous lung resection cohorts, reported concordant effect estimates, and yielded a pooled Hartung–Knapp interval that remained above unity; nevertheless, the small number of studies and the use of study-specific, non-validated cut-offs precluded a higher rating. Taken together, these certainty ratings indicate that the present findings should be interpreted as exploratory and hypothesis-generating rather than as definitive evidence supporting the routine standalone use of NLR or SII, or any current biomarker threshold, for perioperative risk stratification.

**Table 4:** Summary of findings and certainty of evidence for the main biomarker–outcome analyses.

Biomarker/analysis	Outcome	Studies, n	Participants, n	Hartung–Knapp pooled OR (95% CI)	Common-effect OR (95% CI)	I^2^	Certainty of evidence (GRADE)	Main reasons for downgrading
NLR, continuous (per 1-unit increment)	Postoperative pneumonia	2	1,370	1.90 (0.35–10.45)	1.90 (1.61–2.24)	60.4%	Very low	Serious imprecision (k = 2, very wide Hartung–Knapp CI crossing unity); risk-of-bias concerns from retrospective design; inconsistency
SII, continuous (per 10^2^-unit increment)	Postoperative pneumonia	3	2,184	1.17 (0.86–1.59)	1.12 (1.07–1.17)	76.4%	Very low	Serious imprecision (CI crossing unity); substantial heterogeneity; additional uncertainty from scale harmonization and reconstructed standard errors; retrospective design
NLR, categorical (high vs. low)	PPCs or study-defined postoperative complications	2	282	3.98 (0.79–19.96)	3.98 (1.91–8.31)	0.0%	Very low	Serious imprecision (k = 2, very wide Hartung–Knapp CI crossing unity); indirectness (one study used a broader mixed complication composite); retrospective design
SII, categorical (high vs. low)	Postoperative pneumonia or PPCs	2	590	2.53 (1.04–6.17)	2.53 (1.42–4.53)	0.0%	Low	Imprecision (k = 2); study-specific ROC/Youden-derived cut-offs without external validation; retrospective design

CI, confidence interval; GRADE, Grading of Recommendations, Assessment, Development and Evaluations; NLR, neutrophil-to-lymphocyte ratio; OR, odds ratio; PPCs, postoperative pulmonary complications; ROC, receiver operating characteristic; SII, systemic immune-inflammation index.

The Hartung–Knapp pooled OR represents the prespecified primary estimate; the common-effect (inverse-variance) OR is reported as a robustness check. Categorical analyses were considered secondary and exploratory because cut-offs were study-specific and not externally validated.

## Discussion

This systematic review and meta-analysis represents, to our knowledge, the first quantitative synthesis specifically focused on the prognostic associations of preoperative NLR and SII—two routinely available circulating immune-inflammatory biomarkers—with postoperative pneumonia and pulmonary complications after lung resection or esophagectomy. Across both continuous and categorical analytical layers, higher preoperative NLR and SII showed directionally consistent associations with an increased risk of postoperative infectious pulmonary outcomes or closely related postoperative complication composites. For NLR, the continuous point estimate approximated a twofold increase in the odds of postoperative pneumonia per one-unit increment (common-effect OR 1.90; Hartung–Knapp OR 1.90, 95% CI 0.35–10.45), and categorical analyses suggested an approximately fourfold higher odds among patients classified above study-specific thresholds. For SII, the continuous association was more modest in magnitude (common-effect OR 1.12 per 10^2^ units), while categorical analyses indicated more than twice the odds among patients classified as high SII according to study-specific cut-offs; the categorical SII analysis was the only one in which the conservative Hartung–Knapp confidence interval did not cross unity. Although the direction of effect was consistent across all individual studies and both biomarkers, the evidence base remains limited in size (k = 2–3 per analysis), is composed exclusively of retrospective cohorts predominantly from Chinese-speaking East Asian surgical settings, and is of low to very low certainty; notably, the Hartung–Knapp confidence intervals crossed unity in three of the four pooled analyses. These findings should therefore be interpreted as a directionally coherent but preliminary prognostic signal, suitable for hypothesis generation rather than as definitive evidence for clinical implementation or for establishing clinically validated decision thresholds.

The observed association between elevated preoperative NLR and postoperative pneumonia or related pulmonary complications is biologically plausible and consistent with the established role of NLR as a marker of systemic immune-inflammatory imbalance (8). A higher NLR reflects relative predominance of neutrophil-driven innate inflammation, including the release of reactive oxygen species, proteolytic enzymes, and neutrophil extracellular traps, together with relative suppression of lymphocyte-mediated adaptive immune surveillance during the vulnerable perioperative window (31–33). In patients undergoing thoracic surgery, where direct surgical manipulation of the lung parenchyma, one-lung ventilation, and disruption of bronchial clearance mechanisms already predispose to infectious complications (1, 2), a preoperatively skewed NLR may identify individuals whose baseline immune-inflammatory state renders them more susceptible to postoperative pneumonia. The relatively large per-unit effect magnitude observed for NLR likely reflects, at least in part, the compact numerical scale of this ratio, which amplifies the per-unit odds ratio relative to broader-range indices such as SII, rather than necessarily indicating a stronger underlying biological effect (34). The consistent direction of the NLR association across both lung resection and esophagectomy cohorts—including patients exposed to neoadjuvant chemo-immunotherapy—suggests that the prognostic signal may extend across different thoracic surgical settings; however, with only two studies contributing to the continuous NLR analysis, this observation is preliminary and requires confirmation in larger, more diverse cohorts (35).

The SII extends the conceptual framework of NLR by incorporating platelet count, thereby capturing an additional dimension of the host inflammatory response: platelet-driven thromboinflammation (36, 37). Activated platelets interact with neutrophils and endothelial cells, promote microthrombus formation, and release pro-inflammatory mediators that may amplify tissue injury at sites of surgical trauma (38). Conceptually, SII may therefore provide a more comprehensive snapshot of the perioperative immune-inflammatory milieu than NLR alone (8–10). The present review found that higher preoperative SII was directionally associated with increased postoperative pneumonia risk across all contributing studies, and the categorical analysis yielded a pooled OR of 2.53, although this estimate was based on only two studies and the absence of detectable heterogeneity should be interpreted cautiously. However, the continuous per 100-unit effect estimate was more modest (common-effect OR 1.12) than for NLR. Several factors may explain this apparent discrepancy. First, SII values span a substantially wider numerical range than NLR, typically from several hundred to well over one thousand, so that a 100-unit increment represents a proportionally smaller shift along the biomarker distribution and is expected to yield a per-increment OR closer to unity. Second, between-study heterogeneity in SII parameterization was more pronounced than for NLR, with original studies reporting effects per 1 unit, per 100 units, or per standard deviation, necessitating rescaling and, in two cases, reconstruction of standard errors from statistically coherent confidence bounds because of probable typographical errors in the original reports; these data-handling steps, while methodologically justified and transparently documented, introduce additional analytical uncertainty. Third, the SII literature in the thoracic surgical context remains at an early stage, and the small number of available studies (k = 3 for continuous, k = 2 for categorical) limits the precision of summary estimates. Accordingly, the present findings should not be interpreted as evidence that SII lacks prognostic value, but rather that the current evidence base is insufficient to precisely quantify its incremental contribution beyond NLR. Whether SII offers prognostic information independent of NLR—or whether the two indices are largely collinear in this clinical setting—remains an important question that only studies reporting both biomarkers within the same multivariable model can adequately address.

A deliberate methodological feature of the present review was the hierarchical analytical framework that prioritized continuous biomarker associations as the primary synthesis layer and treated categorical high-versus-low comparisons as secondary. This design decision was motivated by a well-recognized problem in the prognostic biomarker literature: the extreme heterogeneity in dichotomization thresholds across studies (39, 40). Even within the small evidence base identified here, NLR cut-offs ranged from 2.30 to 3.0 and SII cut-offs from 261 to 320, all derived from study-specific ROC/Youden index optimization. Such data-driven thresholds are inherently sample-dependent, prone to overfitting, and unlikely to be directly transferable across populations with different baseline inflammatory profiles, surgical case mixes, and outcome rates (41, 42). By analyzing biomarker effects on a continuous scale, the primary synthesis circumvented this source of between-study incomparability and preserved the full information content of the exposure variable, yielding estimates that describe the incremental change in risk per unit increase in the biomarker rather than the contrast between arbitrarily defined extreme groups (39). The categorical results were directionally concordant with the continuous findings for both NLR and SII, which supports the consistency of the observed prognostic signal. At the same time, the categorical effect sizes were predictably larger in magnitude—approximately fourfold for NLR and twofold for SII—because dichotomization inherently compares the tails of the biomarker distribution rather than capturing gradual, dose-dependent risk. This pattern is a well-described statistical artifact of dichotomization and should not be mistaken for a stronger biological signal (40). From a clinical translation perspective, however, categorical thresholds remain attractive because they offer a simple, potentially actionable decision framework, for example by flagging patients above a study-specific inflammatory threshold for closer perioperative pulmonary surveillance. The challenge, as highlighted by the present data, is that no single validated cut-off has yet emerged for either biomarker in the thoracic surgical setting, and the available thresholds should be regarded as study-specific rather than ready for clinical application. Future efforts to establish clinically useful thresholds would benefit from large, prospective derivation–validation cohort designs rather than continued reliance on study-specific ROC optimization (42).

This review has several methodological strengths, including prospective protocol registration, PRISMA 2020 reporting, use of the QUIPS tool for risk of bias assessment, a hierarchical synthesis strategy that prioritized continuous associations to circumvent cut-off heterogeneity, strict within-type pooling of effect measures, and a comprehensive search strategy encompassing seven electronic databases, including Chinese-language databases. Nevertheless, these strengths must be weighed against important limitations arising from both the primary evidence base and the analytical constraints it imposed. The most fundamental limitation is the small number of studies contributing to each meta-analysis (k = 2–3), which substantially limits statistical power, precludes meaningful meta-regression or subgroup analysis, and renders the Hartung–Knapp adjustment so conservative that confidence intervals crossed unity in three of the four pooled analyses. All included studies were retrospective in design, introducing inherent risks of selection bias, information bias, and residual confounding that cannot be fully mitigated by multivariable adjustment (42). The geographic concentration of the evidence—seven of eight studies from Chinese-speaking East Asian settings—raises concerns about generalizability to other ethnic, genetic, and healthcare-system contexts. Several sources of clinical heterogeneity further complicate interpretation. The included studies spanned two distinct surgical populations—lung resection and esophagectomy—that differ in baseline complication rates, operative physiology, and patient demographics. Within the esophagectomy subgroup, two studies enrolled patients who had received neoadjuvant chemoradiotherapy or chemo-immunotherapy, treatments known to alter baseline inflammatory and immune parameters and potentially modify the prognostic value of preoperative biomarkers (35). Whether elevated NLR or SII in neoadjuvant-treated patients reflects tumor-driven inflammation, treatment-induced immune perturbation, or both could not be disentangled. Outcome definitions were not standardized; among the studies contributing to the categorical synthesis, one used a broader mixed complication composite incorporating non-pulmonary events, while another used a pulmonary-specific composite rather than isolated postoperative pneumonia (43). The depth of multivariable adjustment varied substantially, ranging from as few as two covariates to comprehensive risk scores such as ARISCAT; residual confounding from unmeasured factors—including pulmonary function, nutritional status, smoking history, and comorbidity burden—cannot be excluded. Additional analytical limitations included the need for standard-error reconstruction in two SII studies and *p*-value–derived standard-error estimation in a third, reflecting suboptimal reporting quality in the primary literature. Publication bias could not be formally assessed owing to insufficient study numbers, and the predominance of positive findings among small studies predominantly from Chinese-speaking East Asian settings raises the possibility that null or negative results may be underrepresented (44). Finally, no included study directly compared the prognostic performance of NLR versus SII within the same model, preventing any conclusion about the incremental value of one index over the other.

The present findings are best interpreted within the broader and rapidly expanding literature on systemic inflammation-based biomarkers in thoracic surgery, which extends well beyond NLR and SII to include the C-reactive protein-to-albumin ratio, the systemic inflammation response index, and various platelet-based composite indices. Recent work has examined these markers not only in the context of postoperative pneumonia but also across other clinically important endpoints, including severe postoperative complications and perioperative mortality. For example, systemic inflammation-based indices have been evaluated for their ability to predict mortality in post-pneumonectomy bronchopleural fistula (11), illustrating their potential relevance to high-acuity surgical complications beyond infectious pulmonary outcomes. Equally important is the distinction between static and dynamic assessment: whereas the present review focused exclusively on a single preoperative measurement, emerging evidence suggests that the perioperative trajectory of inflammatory indices—rather than a fixed baseline value—may carry additional prognostic information, as has been explored in a related thoracic surgical risk-stratification setting, such as first-episode primary spontaneous pneumothorax (12). Situating preoperative NLR and SII within this wider framework underscores two points relevant to the present synthesis: first, that no single inflammatory index is likely to be optimal across all thoracic surgical populations and outcomes; and second, that future research should consider both static baseline values and dynamic perioperative changes, ideally comparing multiple indices within unified prediction frameworks rather than evaluating each marker in isolation.

Despite these limitations, the present findings carry preliminary clinical implications if interpreted cautiously. Both NLR and SII are derived from routine preoperative complete blood counts, requiring no additional laboratory costs, specialized equipment, or extended turnaround time. This accessibility makes them attractive candidates for integration into perioperative risk stratification frameworks, particularly in resource-limited settings where advanced immunological profiling or costly biomarker panels are unavailable. In practical terms, an elevated preoperative NLR or SII could potentially serve as a simple, early warning signal to prompt targeted perioperative pulmonary risk-mitigation strategies—such as chest physiotherapy, early mobilization protocols, preventive respiratory interventions, or closer postoperative surveillance for infectious complications—in patients identified as higher risk before surgery. However, the current evidence does not support the adoption of any specific NLR or SII threshold for clinical decision-making, and these biomarkers should not be used in isolation but rather considered alongside established risk assessment tools such as the ARISCAT score, pulmonary function testing, nutritional status evaluation, and frailty screening. To move from the directional prognostic signal identified here toward clinically actionable evidence, several research priorities can be identified. First, prospective, multicenter cohort studies with adequate sample sizes are needed to confirm the associations observed in the present retrospective evidence base and to provide more precise effect estimates. Second, future studies should adopt standardized outcome definitions for postoperative pneumonia and pulmonary complications—ideally aligned with international consensus criteria—to reduce the heterogeneity that currently hampers evidence synthesis. Third, studies reporting both NLR and SII within the same multivariable model are essential to determine whether these indices provide independent or largely overlapping prognostic information. Fourth, derivation–validation study designs should replace the current reliance on study-specific ROC-optimized cut-offs, which are unlikely to be reproducible across populations. Fifth, the interaction between neoadjuvant oncologic therapy and preoperative immune-inflammatory biomarker profiles deserves specific investigation, given the growing use of chemo-immunotherapy in thoracic malignancies and its potential to alter the prognostic landscape. Finally, the integration of NLR and SII with existing clinical prediction models and emerging perioperative biomarkers—including C-reactive protein, procalcitonin, and albumin-based nutritional indices—warrants exploration to determine whether composite immune-inflammatory–nutritional panels offer superior risk discrimination compared with any single index.

## Conclusion

This systematic review and meta-analysis provides preliminary pooled evidence that higher preoperative NLR and SII may be associated with an increased risk of postoperative pneumonia and pulmonary complications following thoracic surgery. The direction of effect was generally consistent across continuous and categorical analytical frameworks, across the available lung resection and esophagectomy studies, and across both biomarkers. However, the evidence base remains small (k = 2–3 per analysis), exclusively retrospective, and geographically concentrated, with Hartung–Knapp confidence intervals crossing unity in three of the four pooled analyses and an overall certainty of evidence rated as low to very low. These circulating immune-inflammatory indices therefore represent promising, low-cost candidate prognostic markers whose role should be regarded as hypothesis-generating; their value for routine perioperative risk stratification will require confirmation in larger, prospective, and methodologically standardized studies before clinical adoption can be considered.

## Data Availability

The original contributions presented in the study are included in the article/Supplementary material, further inquiries can be directed to the corresponding author.
