# Supplementary material for: Preoperative neutrophil-to-lymphocyte ratio and systemic immune-inflammation index as prognostic biomarkers for postoperative pneumonia and pulmonary complications after thoracic surgery: a systematic review and meta-analysis
Source: Front Med (Lausanne). 2026 Jun 5;13:1867440. doi: 10.3389/fmed.2026.1867440 (PMC13281584; doi:10.3389/fmed.2026.1867440)
Supplement: Supplementary file 1 [file Data_Sheet_1.PDF]

Supplementary Appendix S1. Electronic Database Search Strategies

All databases were searched from inception to 11 April 2026. No restrictions on language, publication date, or country of origin were applied.

1. PubMed/MEDLINE

("neutrophil-to-lymphocyte ratio"[tiab] OR "neutrophil to lymphocyte ratio"[tiab] OR NLR[tiab]
OR "systemic immune-inflammation index"[tiab] OR "systemic immune inflammation index"[tiab] OR SII[tiab])
AND
(lung resection[tiab] OR pulmonary resection[tiab] OR lobectomy[tiab] OR segmentectomy[tiab]
OR pneumonectomy[tiab] OR "wedge resection"[tiab] OR esophagectomy[tiab] OR oesophagectomy[tiab]
OR thoracic surg*[tiab] OR thoracoscop*[tiab] OR VATS[tiab] OR "video-assisted thorac*"[tiab])
AND
(postoperative[tiab] OR perioperative[tiab] OR pneumonia[tiab] OR "pulmonary complication*"[tiab]
OR "respiratory complication*"[tiab] OR "postoperative complication*"[tiab] OR morbidity[tiab])
Records retrieved: 110

2. Embase (Embase.com)

('neutrophil to lymphocyte ratio':ti,ab OR 'neutrophil-to-lymphocyte ratio':ti,ab OR nlr:ti,ab
OR 'systemic immune inflammation index':ti,ab OR 'systemic immune-inflammation index':ti,ab OR sii:ti,ab)
AND
('thoracic surgery'/exp OR 'lung resection'/exp OR 'esophagectomy'/exp
OR pulmonary resection:ti,ab OR lobectomy:ti,ab OR segmentectomy:ti,ab OR pneumonectomy:ti,ab
OR 'wedge resection':ti,ab OR vats:ti,ab OR thoracoscop*:ti,ab OR 'video assisted thorac*':ti,ab)
AND
(postoperative:ti,ab OR perioperative:ti,ab OR pneumonia/exp OR pneumonia:ti,ab
OR 'pulmonary complication*':ti,ab OR 'respiratory complication*':ti,ab
OR 'postoperative complication*':ti,ab OR morbidity:ti,ab)
Records retrieved: 115

3. Web of Science Core Collection

TS=(("neutrophil-to-lymphocyte ratio" OR "neutrophil to lymphocyte ratio" OR NLR
OR "systemic immune-inflammation index" OR "systemic immune inflammation index" OR SII)
AND

("lung resection" OR "pulmonary resection" OR lobectomy OR segmentectomy OR pneumonectomy
OR "wedge resection" OR esophagectomy OR oesophagectomy OR "thoracic surg*" OR VATS
OR thoracoscop* OR "video-assisted thorac*")
AND
(postoperative OR perioperative OR pneumonia OR "pulmonary complication*"
OR "respiratory complication*" OR "postoperative complication*" OR morbidity))
Records retrieved: 17

4. Scopus
TITLE-ABS-KEY(("neutrophil-to-lymphocyte ratio" OR "neutrophil to lymphocyte ratio" OR NLR
OR "systemic immune-inflammation index" OR "systemic immune inflammation index" OR SII)
AND
("lung resection" OR "pulmonary resection" OR lobectomy OR segmentectomy OR pneumonectomy
OR "wedge resection" OR esophagectomy OR oesophagectomy OR "thoracic surg*" OR VATS
OR thoracoscop* OR "video-assisted thorac*")
AND
(postoperative OR perioperative OR pneumonia OR "pulmonary complication*"
OR "respiratory complication*" OR "postoperative complication*" OR morbidity))
Records retrieved: 199

5. Cochrane Central Register of Controlled Trials (CENTRAL)
("neutrophil-to-lymphocyte ratio" OR NLR OR "systemic immune-inflammation index" OR SII)
AND
(thoracic OR "lung resection" OR lobectomy OR pneumonectomy OR esophagectomy OR VATS)
AND
(postoperative OR pneumonia OR complication*)
Records retrieved: 22

6. China National Knowledge Infrastructure (CNKI)
SU=(("中性粒细胞淋巴细胞比值" OR "中性粒细胞/淋巴细胞比值" OR NLR
OR "系统性免疫炎症指数" OR "系统免疫炎症指数" OR SII)
AND
("胸外科" OR "肺切除" OR "肺叶切除" OR "肺段切除" OR "全肺切除" OR "楔形切除"
OR "食管切除" OR "胸腔镜" OR VATS)
AND

("术后" OR "围术期" OR "肺炎" OR "肺部并发症" OR "呼吸并发症" OR "并发症"))
Records retrieved: 0

7. Wanfang Data (万方数据)
Search terms equivalent to the CNKI strategy above were used.
Records retrieved: 0
Total records identified: 463

Study	D1: Study Participation	D2: Study Attrition	D3: PF Measurement	D4: Outcome Measurement	D5: Study Confounding	D6: Statistical Analysis & Reporting	Rationale / Key Methodological Notes
**Miao, 2024**	Low	Low	Low	Low	Moderate	Low	**D4:** Strict pneumonia criteria based on clinical/imaging/lab data. **D5:** Multivariable model missed core covariates (age, smoking) because they were excluded after univariate screening (p>0.1).
**Lo, 2024**	Low	Low	Low	Moderate	Moderate	Moderate	**D4:** Composite PPCs included non-infectious events (e.g., pneumothorax). **D5:** Adjusted only for clinical T stage; major unadjusted confounders remain. **D6:** OR was mislabeled as HR in the original text.
**Shi, 2022**	Low	Low	Low	High	Moderate	Moderate	**D4:** Extremely broad composite outcome (mixed with anastomotic leakage, DVT, etc.), lowering specificity for infectious events. **D5:** Adjusted only for BMI and p-Stage, lacking age and smoking.
**Shen, 2017**	Low	Low	Low	Low	Moderate	Low	**D4:** Clear criteria for pneumonia (≥3 clinical signs). **D5:** Adjusted for CCI (which partially controls for comorbidities) and lung function, but lacked explicit adjustment for age and smoking history.
**Ding, 2025**	Low	Low	Low	Low	Low	Low	**D4:** Strict CDC criteria for pneumonia. **D5:**

Study	D1: Study Participation	D2: Study Attrition	D3: PF Measurement	D4: Outcome Measurement	D5: Study Confounding	D6: Statistical Analysis & Reporting	Rationale / Key Methodological Notes
							Excellent confounding control by incorporating the ARISCAT score, which inherently adjusts for age, preoperative SpO2, and respiratory infection history.
**Mao, 2022**	Low	Low	Low	Moderate	Moderate	Moderate	**D4:** PPCs composite based on ERAS guidelines. **D5:** Adjusted for surgical extent and lung function, but missed age/smoking. **D6:** Potential logical mismatch between the reported 95% CI and P-value.
**de Fréminville, 2024**	Low	Moderate	Low	Moderate	High	Moderate	**D2:**a substantial proportion of otherwise eligible patients were excluded from the final analysis due to missing perioperative hematologic data.**D4:** Used Clavien-Dindo for major complications but mixed pulmonary with CV/neuro events. **D5:** Inflammatory indices were eliminated during multivariable modeling; only crude, unadjusted estimates are available.
**Jiang, 2023**	Low	Low	Low	Low	Moderate	Moderate	**D4:** Clear pneumonia definition. **D5:** Multivariable regression retained only BMI and SII, severely omitting key clinical confounders (age, surgery type, lung

Study	D1: Study Participation	D2: Study Attrition	D3: PF Measurement	D4: Outcome Measurement	D5: Study Confounding	D6: Statistical Analysis & Reporting	Rationale / Key Methodological Notes
							function). **D6:** Use of original SII units yielded a tiny OR (1.001).

Supplementary Table S1. Risk of bias assessment of included studies using the Quality In Prognosis Studies (QUIPS) tool. Each domain was independently rated by two reviewers as low, moderate, or high risk of bias. D1, study participation; D2, study attrition; D3, prognostic factor measurement; D4, outcome measurement; D5, study confounding; D6, statistical analysis and reporting. The rightmost column provides the key methodological rationale underlying each non-low judgment.

Study ID	Biomarker	Original OR	Original 95% CI	Original unit increment	Rescaled OR	Rescaled 95% CI	SE derivation method	Data handling note	Adjustment status	Covariates in model
**Shen, 2017**	NLR	2.171	1.721–2.737	per 1-unit	2.171	1.721–2.737	Derived from published 95% CI on log scale	None (used as reported)	Adjusted	ppoFEV1%, Charlson comorbidity index (CCI) score >3, ASA score >3, ppoDlco%, NLR
**Ding, 2025**	NLR	1.66	1.31–2.10	per 1-unit	1.66	1.31–2.10	Derived from published 95% CI on log scale	None (used as reported)	Adjusted	ARISCAT score, NLR, SII
**de Fréminville, 2024**	NLR	1.25	0.98–1.59	per 1 SD	N/A	N/A	Derived from published 95% CI on log scale	Retained for qualitative summary only; excluded from primary synthesis due to lack of multivariable adjustment and use of standard deviation (SD) increments.	Unadjusted	None (univariate analysis only)
**Miao, 2024**	SII	1.38	1.19–2.83	per 100-unit	1.38	1.19–1.60	Derived from lower CI on log	Upper CI mathematically	Adjusted	Coronary heart disease, Type of

Study ID	Biomarker	Original OR	Original 95% CI	Original unit increment	Rescaled OR	Rescaled 95% CI	SE derivation method	Data handling note	Adjustment status	Covariates in model
							scale	corrected from 2.83 to 1.60 due to severe logistical asymmetry indicating a typographical error in the original publication.		surgery, Total fluids, Preoperative prealbumin, Preoperative SII
**Ding, 2025**	SII	1.09	1.02–1.71	per 100-unit	1.09	1.02–1.17	Derived from lower CI on log scale	Upper CI mathematically corrected from 1.71 to 1.17 due to severe logistical asymmetry indicating a typographical error in the original publication.	Adjusted	ARISCAT score, NLR, SII
**Jiang, 2023**	SII	1.001	1.000–1.001	per 1 raw unit	1.11	1.05–1.17	Derived from published p-value	Rescaled from per-unit to per	Adjusted	BMI, SII

Study ID	Biomarker	Original OR	Original 95% CI	Original unit increment	Rescaled OR	Rescaled 95% CI	SE derivation method	Data handling note	Adjustment status	Covariates in model
							(p < 0.001)	100-unit increment via exponentiation ($OR = 1.001^{100}$). SE derived from reported p-value due to insufficient decimal precision in the published CI.		
**de Fréminville, 2024**	SII	1.21	0.96–1.54	per 1 SD	N/A	N/A	Derived from published 95% CI on log scale	Retained for qualitative summary only; excluded from primary synthesis due to lack of multivariable adjustment and use of standard deviation (SD) increments.	Unadjusted	None (univariate analysis only)

Supplementary Table S2. Study-level extracted effect estimates for continuous biomarker associations. Original values are reported as published; rescaled

and corrected values reflect standardization to per 1-unit (NLR) or per 10^2 units (SII) and statistical correction of probable typographical errors in published confidence intervals (see Methods, Section 2.6). Standard error derivation methods are documented for transparency.

Study ID	Biomarker	Cut-off value	Cut-off derivation method	AUC (95% CI)	Sensitivity / Specificity	High group n/N	Low group n/N	Outcome	Outcome window	OR (95% CI)	Adjustment status	Covariates in model
**Lo, 2024**	NLR	3	ROC/Youden	0.658 (0.551–0.765)	57.1% / 70.8%	12/45	9/66	PPCs	30d	3.564 (1.344–9.456)	Adjusted	Clinical T classification (T4)
**Shi, 2022**	NLR	2.30	ROC/Youden	0.638 (0.525–0.751)	68.3% / 61.5%	28/78	13/93	complications	in-hospital	4.605 (1.501–14.126)	Adjusted	BMI, p-Stage
**Miao, 2024**	SII	261	ROC/Youden	0.69 (0.65–0.75)	78.3% / 42.8%	NR	NR	Pneumonia	7d	2.40 (1.15–5.02)	Adjusted	Coronary heart disease, Type of surgery, Total fluids
**Mao, 2022**	SII	320.22	ROC/Youden	0.612 (0.541–0.679)	81.3% / 43.1%	26/114	6/90	PPCs	1 month	2.773 (1.075–7.159)	Adjusted	Surgery method, WBC, CRP, number of segments removed, FEV1%, DLCO%, ICU stay

Supplementary Table S3. Study-level extracted effect estimates and cut-off characteristics for categorical biomarker associations (high vs low). Cut-off derivation methods, diagnostic performance metrics (where available), and event rates are reported to facilitate interpretation of between-study heterogeneity in dichotomization approaches. **Abbreviations:** NLR, neutrophil-to-lymphocyte ratio; SII, systemic immune-inflammation index; PPCs, postoperative pulmonary complications; OR, odds ratio; CI, confidence interval; AUC, area under the receiver operating characteristic curve; NR, not reported.
